# Adding Manual Therapy to a Respiratory-Rehabilitation Program in Patients with Respiratory Disorders: A Systematic Review and Meta-Analysis with a Meta-Regression

**DOI:** 10.1016/j.jcm.2025.09.003

**Published:** 2025-10-10

**Authors:** Ferran Cuenca-Martínez, Núria Sempere-Rubio, Enrique Carrasco-González, Francisco M. Martínez-Arnau

**Affiliations:** a Department of Physiotherapy, University of Valencia, Valencia, Spain; b Departamento de Fisioterapia, Universidad Europea Miguel de Cervantes, Valladolid, Spain

**Keywords:** Manual Therapy, Respiratory disturbances, Respiratory function, Functional capacity

## Abstract

**Objective:**

The main aim of this study was to assess the effects of adding MT to a Respiratory Rehabilitation (RR) program in patients with Respiratory Disorders (RD).

**Methods:**

A systematic search was carried out in PubMed, EMBASE, CINAHL, PEDro and Google Scholar (February 1^st^ 2023). The outcome measures were pulmonary function parameters (FEV_1_ and FVC); and exercise capacity, through the 6-minute walk test (6-MWT). Standardized mean differences (SMDs) and 95% confidence intervals were calculated and were pooled in a meta-analysis with a random effects model.

**Results:**

In total, 6 studies were included. Regarding the pulmonary function parameters, no significant effects were found in favor of adding MT to a RR program. However, the meta-analysis showed a significant improvement of 6-MWT when MT were added to a RR with a small clinical effect (n = 6, SMD = –0.55 (–1.01 to –0.09)). Lastly, the meta-regression analysis revealed that the intervention time of MT was not significantly correlated with the improvement of FEV1 (R2 = 52.49%, *P = .*16), FVC (R2=4.22%, *P = .*70), or 6-MWT (R^2^=9.95%, *P = .*62).

**Conclusions:**

There is low-quality evidence that adding MT to an RR program can improve the exercise capacity in patients with RD. However, MT plus RR showed no effect on pulmonary function parameters, also with low-quality evidence.

## Introduction

Manual Therapy (MT)^1^ is a set of clinical tools that uses manual techniques on soft, nervous and osteoarticular tissues such a spinal mobilizations, spinal manipulations, massage techniques, or neurodynamic mobilizations with the aim of improving different clinical variables of interest (i.e.: pain intensity,^2^, ^3^, ^4^ disability,^2^ mechanosensitivity,^2^ range of motion,^4^ physical function,^4^ among others). Traditionally, MT techniques have been applied to patients with neuro-orthopedic disorders with pain or limitation of physical function in general.^1^ However, in recent decades there has been growing interest in the study of the influence of MT on pulmonary function variables. The current evidence is controversial, because there are studies that have shown significant effects of MT on pulmonary function variables and other studies have not found them. For example, Engels & Vemulpad^5^ showed that MT in combination with therapeutic exercise improved pulmonary function parameters in asymptomatic individuals.^5^ Hwangbo et al.^6^ found that thoracic mobilisations, together with stretching, improved all pulmonary function parameters in patients with neck pain. The systematic review conducted by Wearing et al.^7^ evaluated the role of spinal manipulation with or without other interventions for the management of chronic obstructive pulmonary disease (COPD). The authors found that most of the included studies resulted in improvements in pulmonary function. However, some studies have not found significant effects on these variables. For example, Hondras et al.^8^ found that MT in isolation did not result in significant improvements in the management of patients with asthma. Also, Noll et al.^9^ found that the application of different MT techniques worsened outcomes on pulmonary function variables after intervention relative to baseline measures.

Despite this, it is important to stress the role of Respiratory Rehabilitation (RR) programs on patients with Respiratory Disorders (RD). In fact, several systematic reviews with and without meta-analysis have shown that a RR program based on a respiratory muscle training improves several pulmonary function parameters and maximal static pressures in patients with different RD.^10^, ^11^, ^12^, ^13^, ^14^, ^15^ In addition, some studies, such as the 1 conducted by López-de-Uralde-Villanueva et al.^16^ or the 1 performed by Balbás-Álvarez et al.^17^ showed that adding MT, together with a motor control exercise program, to a RR program improved pulmonary function, postural measures and maximal static inspiratory pressure. However, we wondered whether adding only MT techniques to a RR program is able to elicit greater clinical changes than the RR program alone in patients with RD. It was therefore that the main aim of this study was to assess the effects of adding MT to a RR program in patients with RD.

## Methods

This systematic review and meta-analysis was performed according to the Preferred Reporting Items for Systematic Reviews and Meta-analysis (PRISMA) guidelines described by Page et al.^18^ The protocol of this systematic review and meta‐analysis was registered in an international registry prior to starting the review (PROSPERO: CRD42023403324).

### Eligibility Criteria

The selection criteria used in this systematic review and meta-analysis were based on methodological and clinical factors, such as the Population, Intervention, Control, Outcomes, and Study design (PICOS) described by Stone.^19^

#### Population

The participants selected for the studies were patients older than 18 years with any kind of RD. The participants’ gender was irrelevant (meaning, there were no gender-based restrictions imposed).

#### Intervention and Control

The intervention group was to add any MT intervention (such as joint mobilizations, spinal manipulations, or soft tissue techniques) to a standard RR program. All studies that did MT without adding to a RR program were excluded. In addition, all studies that added an extra intervention to MT (such as motor control exercises or aerobic exercise, for example) were also excluded. The comparison group had to contain only a standard RR program. This RR program should be based on therapeutic exercise as the core of the program, to which other interventions could be added, such as education programs, medication, nutritional advice, or psychological support.

#### Outcome Measures

The measures used to assess the results and effects were pulmonary function parameters, through the forced expiratory volume during the first second (FEV_1_) and forced vital capacity (FVC); and exercise capacity through the 6-minute walk test (6-MWT).

#### Study Design

We selected randomised controlled trials (RCTs), randomised parallel-design controlled trials, randomised cross-over trials and prospective controlled clinical trials.

### Information Sources

The search for studies was performed using PubMed (Medline), EMBASE, CINAHL, PEDro and Google Scholar. The last search was run on the 1^st^ of February 2023. We used a validated search filter for retrieving studies on measurement properties in PubMed; the same filter was adapted for all other databases.^20^ In addition, the search was adapted and performed in Google Scholar due to its capacity to search for relevant articles and grey literature.^21^^,^^22^ No restrictions were applied to any specific language as recommended by the international criteria.^23^

### Search strategy

The search strategy combined medical subject headings (MeSH) and non-MeSH terms, adding a Boolean operator (OR and/or AND) to combine them. The terms were as follows: “Breathing Exercises” [MeSH Terms], “inhalation” [MeSH Terms], “musculoskeletal manipulations” [MeSH Terms], (“manipulation, osteopathic” [MeSH Terms], “manipulation,” “mobilization,” “inspiratory muscle training,” or “respiratory muscle training,” among others (See supplementary file Annex 1). Two independent reviewers (X and Y) conducted the search using the same methodology, and the differences were resolved by consensus. Additionally, meticulous manual searches were performed, including journals that have published articles related to the topic of this review as well as reference lists of the included studies. The reference sections of the original studies were screened manually. To remove duplicates, we employed the citation management software Mendeley (Mendeley desktop v1.17.4, Elsevier, New York, New York) and hand-checked the citations.^24^

### Selection Criteria

**Fig 1:**
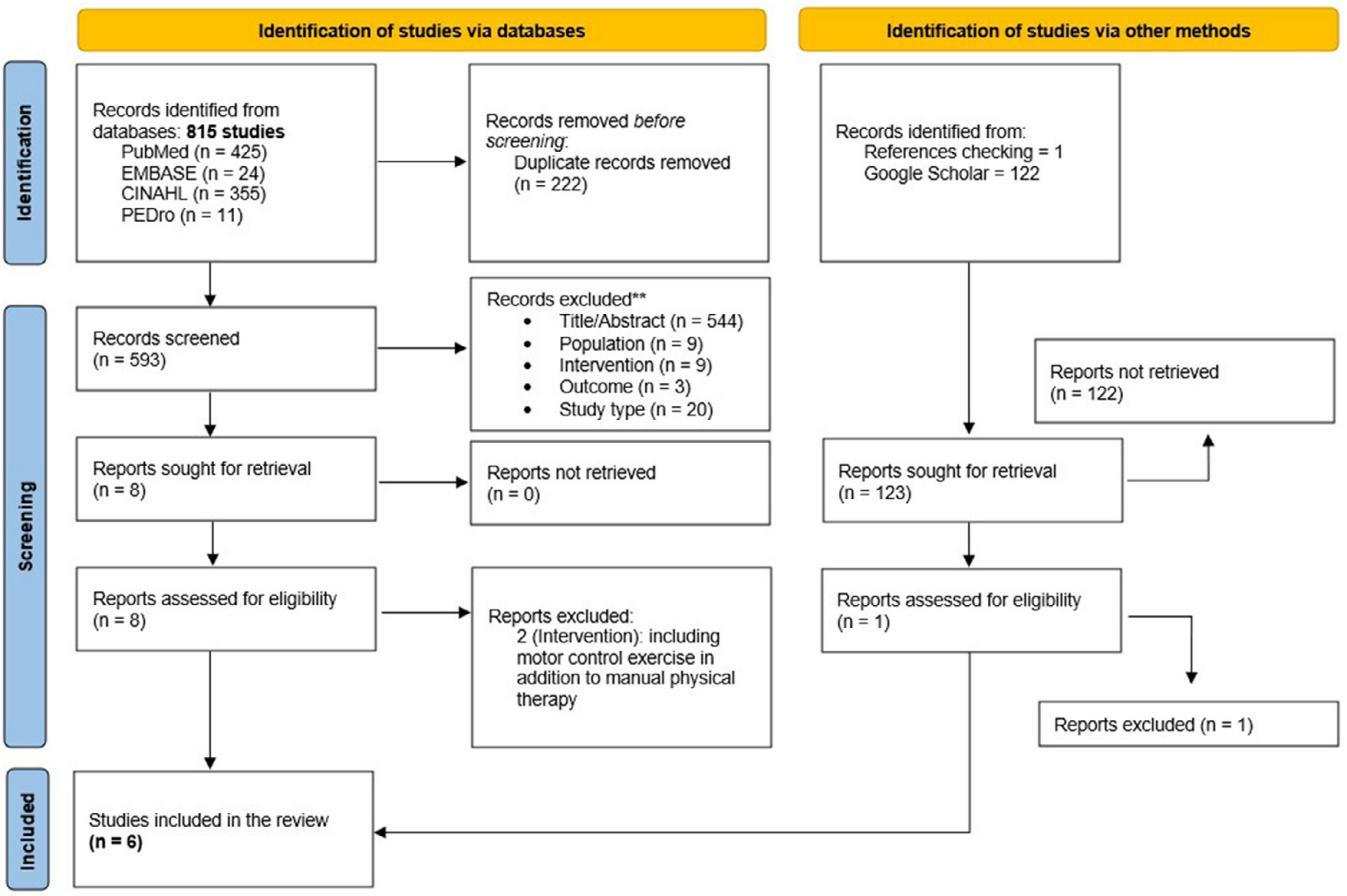
PRISMA Flowchart for selecting studies. *Consider, if feasible to do so, reporting the number of records identified from each database or register searched (rather than the total number across all databases/registers). **If automation tools were used, indicate how many records were excluded by a human and how many were excluded by automation tools. *From:* Page MJ, McKenzie JE, Bossuyt PM, Boutron I, Hoffmann TC, Mulrow CD, et al. The PRISMA 2020 statement: an updated guideline for reporting systematic reviews. BMJ 2021;372:n71. 10.1136/bmj.n71. For more information, visit: http://www.prisma-statement.org/.

First, 2 independent reviewers (X and Y) assessed the relevance of the RCTs concerning the study questions and aims. They screened the papers for relevance using information from the title, abstract, and keywords of each study. If there was no consensus or the abstracts did not contain sufficient information, the full text was reviewed. In the second phase of the analysis, the full text was used to assess whether the studies met all the inclusion criteria (Fig 1). Differences between the 2 independent reviewers were resolved by a consensus process moderated by a third reviewer (Z).^25^

### Data Collection Process

Two independent researchers (X and Y) extracted study characteristics and outcome data using a structured protocol that ensured that the most relevant information was obtained from each study.^26^ The following data were extracted: authors, population, sample size, treatments (intervention and comparator), the outcome measures (FEV_1_, FVC and 6-MWT), and the obtained results.

### Study Risk of Bias Assessment

We used the Cochrane Handbook for Systematic Reviews of Interventions version 5.1.0 to assess the risk of bias in the included studies.^27^ The assessment tool covers a total of 7 domains: (1) random sequence generation (selection bias), (2) allocation concealment (selection bias), (3) blinding of participants and personnel (performance bias), (4) blinding of outcome assessments (detection bias), (5) incomplete outcome data (attrition bias), (6) selective reporting (reporting bias), and (7) other biases. Bias was assessed as low risk, high risk, or unclear risk.

The studies’ methodological quality was assessed using the PEDro scale,^28^ which assesses the internal and external validity of a study and consists of 11 criteria: (1) specified study eligibility criteria, (2) random allocation of patients, (3) concealed allocation, (4) measure of similarity between groups at baseline, (5) patient blinding, (6) therapist blinding, (7) assessor blinding, (8) fewer than 15% dropouts, (9) intention-to-treat analysis, (10) intergroup statistical comparisons, and (11) point measures and variability data. The methodological criteria were scored as follows: yes (1 point), no (0 points), or do not know (0 points). The PEDro score for each selected study provided an indicator of the methodological quality (9-10 = excellent; 6-8 = good; 4-5 = fair; 3-0 = poor).^29^ We used the data obtained from the PEDro scale to map the results of the quantitative analyses. Two independent reviewers (X and Y) examined the quality of all the selected studies using the same methodology. Disagreements between the reviewers were resolved by consensus with a third reviewer (Z). The concordance between the results (inter-rater reliability) was measured using Cohen’s kappa coefficient (κ) as follows: 1) κ > 0.7 indicated a high level of agreement between assessors; 2) κ = 0.5-0.7 indicated a moderate level of agreement; and 3) κ < 0.5 indicated a low level of agreement).^30^

### Effect Measures

To compare the outcomes reported by the studies, we calculated the standardised mean difference (SMD) over time and the corresponding 95% confidence interval (CI) for the continuous variables (FEV_1_, FVC and 6-MWT). The statistical significance of the pooled SMD was examined as Hedges’ *g* to account for a possible overestimation of the true population effect size in the small studies.^31^

### Synthesis Methods

The statistical analysis was conducted using *Meta-Essentials* software (version 1.5).^32^ To compare the outcomes reported by the studies, we calculated the standardised mean difference (SMD) over time and the corresponding 95% confidence interval (CI) for the continuous variables. The statistical significance of the pooled SMD was examined as Hedges’ *g* to account for a possible overestimation of the true population effect size in the small studies.^31^ We used the same inclusion criteria for the systematic review and the meta-analysis and included 3 additional criteria: (1) In the results, there was detailed information regarding the comparative statistical data of the exposure factors, therapeutic interventions, and treatment responses; (2) the intervention was compared with a similar control group; and (3) data on the analysed variables were represented in at least 3 studies. The estimated SMDs were interpreted as described by Hopkins et al.^33^, that is, we considered that an SMD of 4.0 represented an extremely large clinical effect, 2.0-4.0 represented a very large effect, 1.2-2.0 represented a large effect, 0.6-1.2 represented a moderate effect, 0.2-0.6 represented a small effect, and 0.0-0.2 represented a trivial effect. We estimated the degree of heterogeneity among the studies using Cochran’s Q statistic test (a p-value <.05 was considered significant) and the inconsistency index (I^2^).^33^ We considered that an I^2^>25% represented small heterogeneity, I^2^>50% represented medium heterogeneity, and I^2^>75% represented large heterogeneity.^34^ The I^2^ index is a complement to the Q test, although it has the same problems of power with a small number of studies.^34^ When the Q-test was significant (*P* < .1) and/or the result of I^2^ was >75%, there was heterogeneity among the studies, and the random-effects model was conducted in the meta-analysis. To detect publication biases, a visual evaluation of the funnel plot seeking asymmetry, was performed. Lastly, we applied a meta-regression analysis to analyse the relationship between the intervention time of MT and pulmonary function and exercise capacity variables using a random effects model employing the effect size statistic (Hedges’ *g*) of the main outcomes scores to correlate with the intervention time of MT.^32^

### Certainty Assessment

The certainty of evidence analysis was based on classifying the results into levels of evidence according to the Grading of Recommendations, Assessment, Development and Evaluation (GRADE) framework, which is based on 5 domains: study design, imprecision, indirectness, inconsistency, and publication bias.^35^ The assessment of the 5 domains was conducted according to GRADE criteria.^36^^,^^37^ Evidence was categorised into the following 4 levels accordingly: (1) *High quality.* Further research is very unlikely to change our confidence in the effect estimate. All 5 domains are also met: (2) *Moderate quality.* Further research is likely to have an important impact on our confidence in the effect estimate and might change the effect estimate. One of the 5 domains is not met: (3) *Low quality.* Further research is very likely to have a significant impact on our confidence in the effect estimate and is likely to change the estimate. Two of the 5 domains are not met; and (4) *Very low quality.* Any effect estimates highly uncertain. Three of the 5 domains are not met.^36^^,^^37^

## Results

### Study Selection

Out of a total of 937 studies identified, 8 underwent full-text review. After a thorough examination, 2 of them were excluded due to their intervention, which included motor control exercises. Consequently, a total of 6 studies were ultimately included in this review (Fig 1).

### Study Characteristics

All included patients in this systematic review were diagnosed with a RD. Three studies included patients with COPD,^38^, ^39^, ^40^ 1 study included patients with Post-Covid-19 symptoms,^41^ 1 study included patients with Cystic Fibrosis^42^ and finally, the last study included patients with Dysfunctional Breathing.^43^ Table 1 lists the descriptive characteristics of the included studies. In addition, Table 2 lists the MT intervention techniques used in the included studies.

**Table 1:** Characteristics of the Included studies

Study	Population (n)	Intervention/s	Comparator	Outcome measures	Results/Conclusions
Buran Cirak et al.^38^	Patients with COPD (60)	RR + MT (3 days/week for 12 weeks)	RR (IMT: 40% of maximal inspiratory pressure; Twice every day for 12 weeks)	FEV_1_ FVC 6-MWT	Significant differences were found in all analysed variables in favor of RR+MT group.
Engel et al.^39^	Patients with COPD (33)	Arm 1: RR + ST Arm 2: RR + ST + SM (Both, twice a week for 8 weeks)	RR (health education, exercise training and non-intervention phase; 24 weeks)	FEV_1_ FVC 6-MWT	Results showed that adding MT significantly improved exercise capacity but not FEV_1_ and FVC.
Jones et al.^43^	Patients with DB (60)	RR + MT	RR (Education, diaphragmatic control and breathing retraining)	FEV_1_ FVC 6-MWT	Results showed that MT did not provide additional benefit in patients with DB.
Nagy et al.^41^	Patients with Post-Covid-19 Syndrome (52)	RR + MT (18 sessions, 3 sessions/week for 6 weeks)	RR (IMT: 60% of maximal inspiratory pressure; 2 sets of 30 dynamic inspiratory efforts, 3 sessions/week for 6 weeks)	6-MWT	Results showed that adding MT significantly improved exercise capacity.
Swender et al.^42^	Patients with CF (33)	RR + MT (1 session daily for 4 to 7 days)	RR (daily during the hospital stay) + Sham MPT	FEV_1_ FVC	Results did not show a benefit of MT in patients with CF.
Zanotti et al.^40^	Patients with COPD (20)	RR + MT (SM; Once a week for 4 weeks for a total of 4 sessions).	RR (exercise training, educational support, psychological counselling, and nutritional intervention; 5 days/week for 4 weeks)	FEV_1_ FVC 6-MWT	Adding MT to PR could increase exercise capacity in patients with COPD.

MT, Manual Therapy; RR, Respiratory Rehabilitation; IMT, Inspiratory Muscle Training, FEV_1_, Forced Expiratory Volume During the First Second; FVC, Forced Vital Capacity; 6-MWT, Six-Minute Walk Test; ST, Soft Tissue; SM, Spinal Manipulative therapy; DB, Dysfunctional Breathing; CF, Cystic Fibrosis.

The primary studies could have included more variables that, not being of interest to this study, were not included in Table 1.

**Table 2:** Characteristics of the Manual Therapy Interventions

Study	Techniques employed
Buran Cirak et al.^38^	• Suboccipital decompression • Gliding of the cervical vertebral articulations in the anterior/posterior direction • Myofascial release of sternocleidomastoid and trapezius muscles • Gliding of sternoclavicular joint in the anterior/posterior direction • Myofascial release of intercostal muscles and paravertebral muscles • Diaphragmatic release • Rib raising • Mobilization of scapulothoracic joint • Gliding of the thoracic vertebral articulations in the anterior/posterior direction
Engel et al.^39^	**Arm 1** • Gentle Effleurage • Friction • Crossfiber • Friction massage applied to the muscles of the posterior chest wall including the intercostal, serratus posterior and anterior, rhomboid, trapezius, latissimus dorsi, erector spinae, quadratus lumborum, and elevator scapulae muscles. **Arm 2** • Arm 1 techniques plus • Spinal manipulation to the to the thoracic intervertebral, costovertebral, and costotransverse joints
Jones et al.^43^	• Maitland mobilization • Muscle energy techniques • Trigger point therapy • Myofascial release techniques • Diaphragm doming • Rib raising
Nagy et al.^41^	• Manual diaphragm release technique
Swender et al.^42^	• Rib raising • Abdominal diaphragm release • Thoracic inlet myofascial release • Thoracic lymphatic pump • Suboccipital decompression
Zanotti et al.^40^	• “The treatment was completely tailored to suit the needs of the individual”

### Risk of Bias in Studies

**Fig 2:**
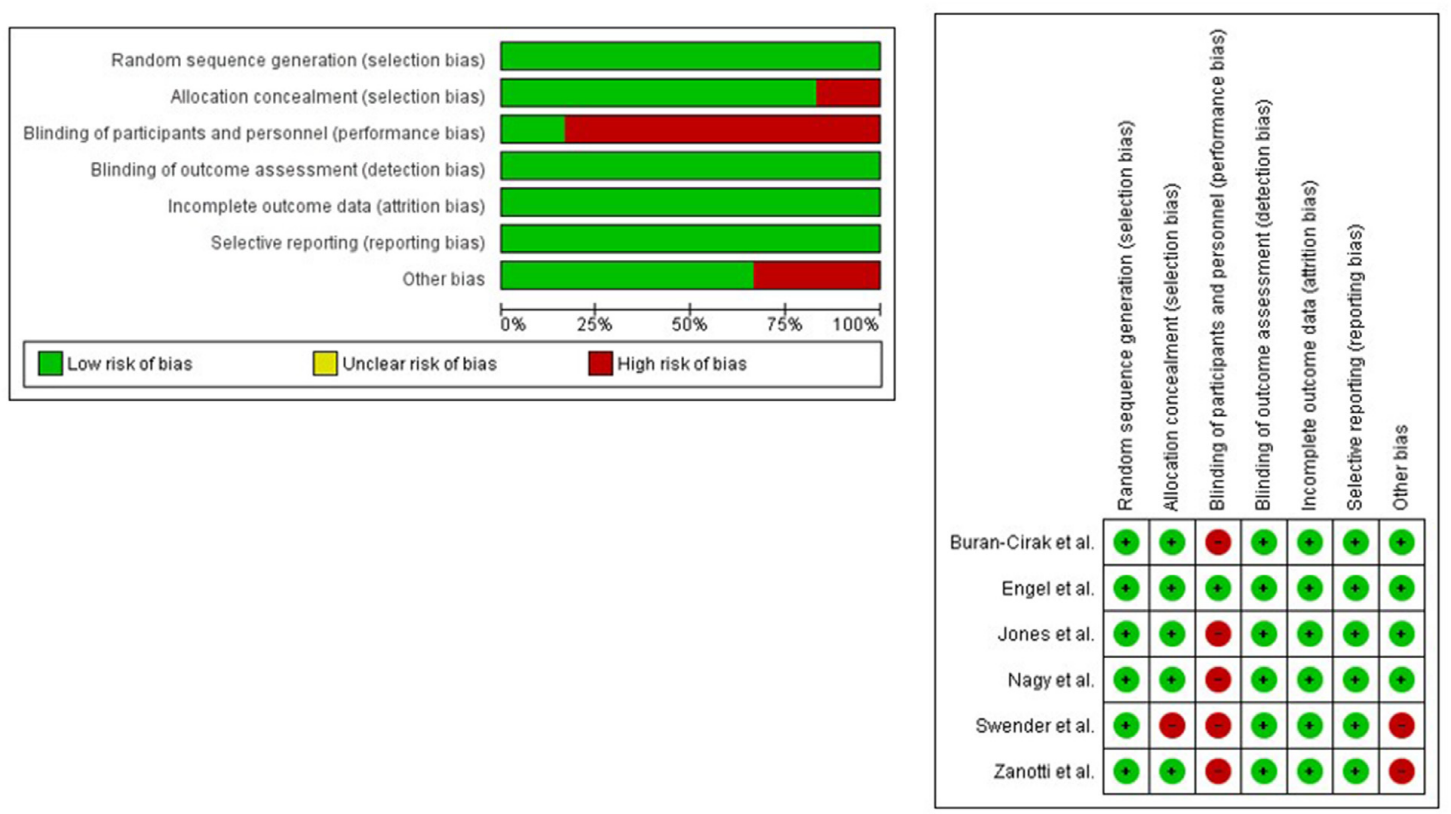
Risk of bias summary. Review authors’ judgements about each risk of bias item for each included study (Risk of Bias scale) and risk of bias graph. Review authors’ judgements about each risk of bias item presented as percentages across all included studies (Risk of Bias scale).

We evaluated the studies’ quality with the Cochrane assessment tool. The 100% of the studies had a low risk of detection bias, attrition bias and reporting bias. The domain with the highest percentage of studies with a high risk of bias was the performance bias. Figure 2 shows the risk of bias summary and risk of bias graph. The inter-rater reliability of the methodological quality assessment was high (κ = 0.796). All of the studies had an excellent or good methodological quality. Table 3 lists the PEDro scores for each study. The inter-rater reliability of the methodological quality assessment between assessors was high (κ=0.87).

**Table 3:** Assessment of the Studies’ Quality Based on the PEDro Scale

Items												
	1	2	3	4	5	6	7	8	9	10	11	Total
Buran Cirak et al.^38^	1	1	1	1	0	0	1	1	1	1	1	**8**
Engel et al.^39^	1	1	1	1	1	0	1	1	1	1	1	**9**
Jones et al.^43^	1	1	1	1	0	0	1	1	1	1	1	**8**
Nagy et al.^41^	1	1	1	1	0	0	1	1	1	1	1	**8**
Swender et al.^42^	1	1	0	1	0	0	1	1	1	1	1	**7**
Zanotti et al.^40^	1	1	1	1	0	0	1	1	1	1	1	**8**

1, patient choice criteria are specified; 2, random assignment of patients to groups; 3, hidden assignment; 4, groups were similar at baseline; 5, all patients were blinded; 6, all therapists were blinded; 7, all evaluators were blinded; 8, measures of at least 1 of the key outcomes were obtained from more than 85% of baseline patients; 9, intention-to-treat analysis was performed; 10, results from statistical intergroup comparisons were reported for at least 1 key outcome; 11, the study provides point and variability measures for at least 1 key outcome.

### Results of Syntheses

#### Pulmonary Function

##### Forced Expiratory Volume During the First Second

**Fig 3:**
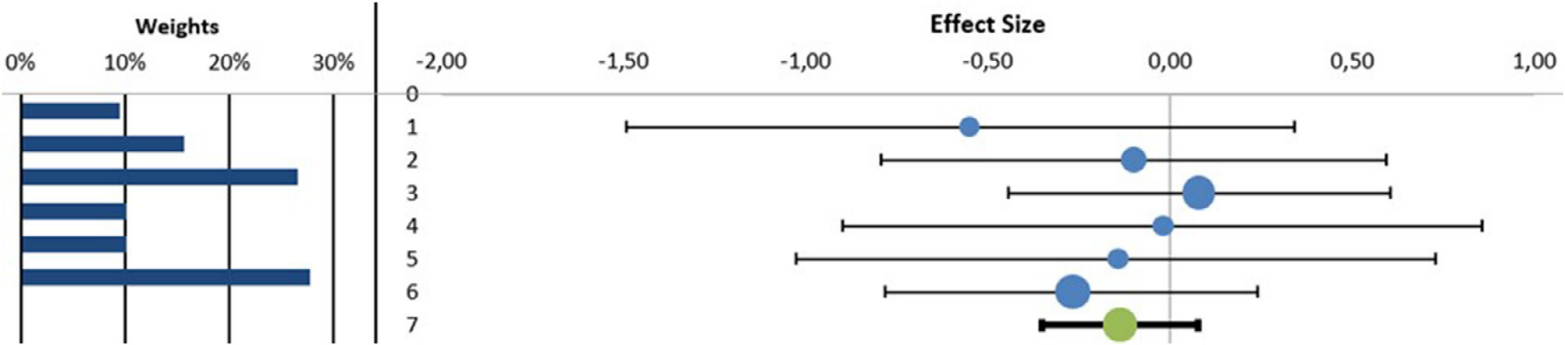
Synthesis forest plot of forced expiratory volume during the first second. The small boxes with the squares represent the point estimate of the effect size and sample size. The lines on either side of the box represent a 95% confidence interval (CI). On the left: in favor of adding manual therapy to respiratory rehabilitation program On the right: in favor of respiratory rehabilitation program Data: Standardized Mean Differences (SMD) (95% CI) –0.14 (–0.35 to 0.08), *P > .*05, I^2^ = 0.0%).

The MA found no statistically significant differences in favor to adding MT to a RR program in the FEV_1_ parameter (n = 6 (216 patients), SMD (95% CI) –0.14 (–0.35 to 0.08), *P* > .05, I^2^ = 0.0%) (Fig 3) with no evidence of significant heterogeneity (Q = 1.93, *P = .*86) (See supplementary file Annex 2). In the meta-regression analysis, we explored the role of the intervention time of MT in improving FEV_1_ parameter. The results showed that the intervention time of MT was not significantly correlated with the improving of FEV_1_ parameter (β = –0.72; *P = .*16 and R^2^ = 52.49%) (See supplementary file Annex 3).

##### Forced Vital Capacity

**Fig 4:**
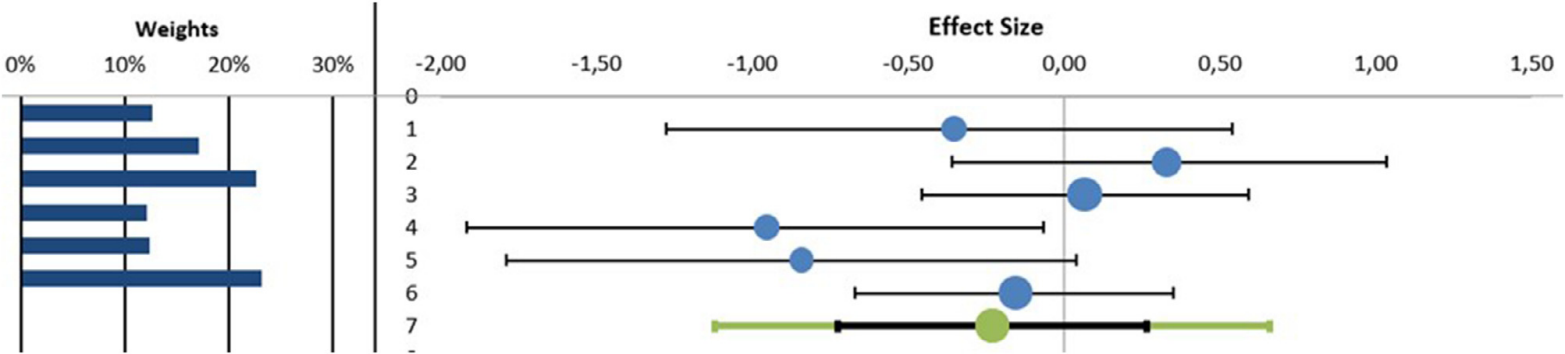
Synthesis forest plot of forced vital capacity. The small boxes with the squares represent the point estimate of the effect size and sample size. The lines on either side of the box represent a 95% confidence interval (CI). On the left: in favor of adding manual therapy to respiratory rehabilitation program On the right: in favor of respiratory rehabilitation program Data: Standardized Mean Differences (SMD) (95% CI) –0.23 (–0.73 to 0.23) *P > .*05, I^2^ = 41.54%).

The MA found no statistically significant differences in favor to adding MT to a RR program in the FVC parameter (n = 6 (216 patients), SMD (95% CI) –0.23 (–0.73 to 0.23) *P > .*05, I^2^ = 41.54%) (Fig 4) with no evidence of significant heterogeneity (Q = 8.55, *P = .*12) (See supplementary file Annex 4). In the meta-regression analysis, we explored the role of the intervention time of MT in improving FVC parameter. The results showed that the intervention time of MT was not significantly correlated with the improving of FVC parameter (β = –0.21; *P = .*70 and R^2^ = 4.22%) (See supplementary file Annex 5).

#### Exercise Capacity

##### Six-Minute Walk Test

**Fig 5:**
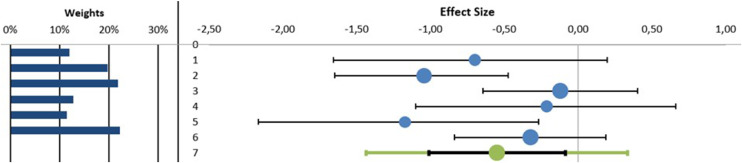
Synthesis forest plot of 6-minute walk test. The small boxes with the squares represent the point estimate of the effect size and sample size. The lines on either side of the box represent a 95% confidence interval (CI). On the left: in favor of adding manual therapy to respiratory rehabilitation program On the right: in favor of respiratory rehabilitation program Data: Standardized Mean Differences (SMD) (95% CI) –0.55 (–1.01 to –0.09) *P < .*05, I^2^ = 43.92%).

The MA found statistically significant differences in favor to adding MT to a RR program in the 6MWT with a small clinical effect (n = 6 (235 patients), SMD (95% CI) –0.55 (–1.01 to –0.09) *P < .*05, I^2^ = 43.92%) (Fig 5) with no evidence of significant heterogeneity (Q = 8.92, *P = .*11) (See supplementary file Annex 6). In the meta-regression analysis, we explored the role of the intervention time of MT in improving 6-MWT variable. The results showed that the intervention time of MT was not significantly correlated with the improving of 6-MWT variable (β = –0.32; *P = .*62 and R^2^ = 9.95%) (See supplementary file Annex 7).

### Certainty of Evidence

According to the GRADE recommendations, there was low-quality of evidence regarding the effects of adding MT to a RR program on all assessed variables (Table 4).

**Table 4:** Summary of Findings and Quality of Evidence (GRADE)

Certainty assessment						No. of participants				
Outcome (No. of Studies)	Study design	Risk of bias	Inconsistency	Indirectness	Imprecision	Intervention	Control	Absolute (95% CI)	Certainty	Importance
*FEV_1_ (6)*	RCT	Not serious	Not serious	Serious	Serious	100	116	−0.14 (−0.35 to 0.08)	**Low** (**+**) (**+**)	Critical
*FVC (6)*	RCT	Not serious	Not serious	Serious	Serious	100	116	−0.23 (−0.73 to 0.23)	**Low** (**+**) (**+**)	Critical
*6-MWT (6)*	RCT	Not serious	Not serious	Serious	Serious	110	125	−0.55 (−1.01 to -0.09)	**Low** (**+**) (**+**)	Critical

FEV1, forced expiratory volume during the firs second; FVC, forced vital capacity; 6-MWT, Six-minute walk test; RCT, randomised controlled trial; CI, confidence interval.

## Discussion

The main aim of this study was to assess the effects of adding MT to a RR program in patients with RD. The results showed that adding MT to a RR program did not result in statistically significant improvements in pulmonary function variables compared to the RR program in isolation. However, we found significant differences with respect to exercise capacity in favor of adding MT to a RR program. Finally, the meta-regression analyses did not show a direct relationship between greater intervention time of MT and greater clinical effect in all the variables studied in the present study.

The findings found in this study agree with those obtained in other similar studies published previously. For example, Simonelli et al.^44^ performed a systematic review to evaluate the role of MT with or without associated therapeutic exercise on some pulmonary function parameters and exercise capacity in patients with COPD. Simonelli et al.^44^ found that MT did not lead to any significant improvement in pulmonary function variables, while the results were mixed with respect to exercise capacity variable. Our results were also similar to those obtained by Heneghan et al.^45^ which found that pulmonary function change minimally after MT and stated that evidence supporting MT is lacking. Ernst^46^ also found similar results in patients with asthma. However, the study conducted by Wearing et al.^7^ found that MT together with therapeutic exercise resulted in significant changes in pulmonary function parameters, although the study by Wearing et al.^7^ only included joint MT (spinal manipulation), unlike our study, the study by Simonelli et al.^44^ and the study by Heneghan et al.^45^ where any MT techniques were included. In addition, our study evaluated the role exclusively of MT without adding any intervention other than RR itself with the aim of isolating the effect of MT.

Finally, in our study we found that adding MT to a RR program resulted in significant improvements in exercise capacity through the 6-MWT compared to only a RR program. In order to justify these findings, we had to review some neurophysiological effects occurring after MT application. Systematic reviews conducted by Wirth et al.^47^ and Kingston et al.^48^ evaluated the effect of MT on the neurovegetative system. Kingston et al.^48^ found that spinal mobilization elicited a consistent activation of the sympathetic-excitatory nervous system, assessing mono-innervated variables, such as skin conductance, or doubly innervated variables, such as heart rate or respiratory rate. In addition to this, Wirth et al.^47^ found similar results in the application of spinal manipulations (high velocity and low amplitude techniques). Moreover, Lascurain-Aguirrebena et al.^49^ found that 1 of the mechanisms of action of MT was sympathoexcitation.

Probably, this sympathetic activity provoked by the application of MT techniques could justify the improvement in exercise capacity. The authors of this study hypothesized that MT could provoke changes in sensorimotor control (muscle function)^49^ of the thoracic spine, and therefore, short-term changes in the recruitment of the musculature involved in breathing. This neurophysiological change could have an impact on breathing and could lead to changes in some parameters of pulmonary function, and this, indirectly, on exercise capacity. However, we did not find changes in pulmonary function parameters, so perhaps the activation of the sympathetic-excitatory system after the application of MT could justify this significant improvement. Thus, it is likely that MT causes some changes in the motor recruitment of the thoracic spine musculature, but not enough to translate into improvements in pulmonary function parameters. However, this is only a hypothesis, and further investigation to explain the associated mechanisms is required.

At the clinical level, therapists might consider adding MT techniques to patients with RR in order to improve exercise capacity. More research is needed before translating these techniques to improving pulmonary function parameters. Perhaps studies combining techniques, comparing application times of the techniques, or comparing them directly against each other may offer more interesting clinical data for the future to see if changes in sensorimotor control may ultimately translate into improvements in pulmonary function parameters.

### Limitations

Firstly, the sample is heterogeneous. This is an important limitation of external validity due to the fact that different patients with different RDs are grouped together. Secondly, the number of articles was small. Even so, this is a first study that can offer preliminary data on the impact of MT added to a RR program in patients with RD. Third, the number of studies included for the meta-regression analysis was low, where in addition, not all studies reported the time of intervention so that they could not be included in the analysis. This should also be considered a limitation. Finally, the set of MT techniques employed in the studies was very heterogeneous. A consensus should be made among Manual Therapists on which techniques could be more indicated with the aim of improving respiratory variables from a neurophysiological point of view.

## Conclusions

In conclusion, there is low-quality of evidence that adding MT to a RR program can improve the exercise capacity in the post-intervention period in patients with RD. However, it seems that MT showed no effect on pulmonary function parameters also with a low-quality of evidence.

## Appendix Supplementary materials

Image, application 1

## Footnotes

Supplementary material associated with this article can be found in the online version at doi:10.1016/j.jcm.2025.09.003.
