# Supplementary material for: Adding Manual Therapy to a Respiratory-Rehabilitation Program in Patients with Respiratory Disorders: A Systematic Review and Meta-Analysis with a Meta-Regression
Source: J Chiropr Med. 2025 Oct 10;24(1-4):361–71. doi: 10.1016/j.jcm.2025.09.003 (PMC12803990; doi:10.1016/j.jcm.2025.09.003)
Supplement: Supplementary file 1 [file mmc1.docx]

**Adding Manual Therapy to a Respiratory-Rehabilitation Program in Patients with Respiratory Disorders: A Systematic Review and Meta-Analysis with a Meta-Regression**

Supplementary file

**Annex 1.** Database search equations.

**PubMed (Medline)**

1-("Breathing Exercises"[MeSH Terms] OR (("inhalation"[MeSH Terms] OR "inhalation"[All Fields] OR "inspiratory"[All Fields]) AND ("muscle s"[All Fields] OR "muscles"[MeSH Terms] OR "muscles"[All Fields] OR "muscle"[All Fields]) AND ("education"[MeSH Subheading] OR "education"[All Fields] OR "training"[All Fields] OR "education"[MeSH Terms] OR "train"[All Fields] OR "train s"[All Fields] OR "trained"[All Fields] OR "training s"[All Fields] OR "trainings"[All Fields] OR "trains"[All Fields])) OR ("Breathing Exercises"[MeSH Terms] OR ("breathing"[All Fields] AND "exercises"[All Fields]) OR "Breathing Exercises"[All Fields] OR ("respiratory"[All Fields] AND "muscle"[All Fields] AND "training"[All Fields]) OR "respiratory muscle training"[All Fields])) AND ("musculoskeletal manipulations"[MeSH Terms] OR ("musculoskeletal"[All Fields] AND "manipulations"[All Fields]) OR "musculoskeletal manipulations"[All Fields] OR ("manual"[All Fields] AND "therapy"[All Fields]) OR "manual therapy"[All Fields] OR ("manipulation, osteopathic"[MeSH Terms] OR ("manipulation"[All Fields] AND "osteopathic"[All Fields]) OR "osteopathic manipulation"[All Fields] OR ("osteopathic"[All Fields] AND "manipulation"[All Fields])) OR ("mobilisation"[All Fields] OR "mobilisations"[All Fields] OR "mobilise"[All Fields] OR "mobilised"[All Fields] OR "mobiliser"[All Fields] OR "mobilisers"[All Fields] OR "mobilises"[All Fields] OR "mobilising"[All Fields] OR "mobilization"[All Fields] OR "mobilizations"[All Fields] OR "mobilize"[All Fields] OR "mobilized"[All Fields] OR "mobilizer"[All Fields] OR "mobilizers"[All Fields] OR "mobilizes"[All Fields] OR "mobilizing"[All Fields]) OR ("mobilisation"[All Fields] OR "mobilisations"[All Fields] OR "mobilise"[All Fields] OR "mobilised"[All Fields] OR "mobiliser"[All Fields] OR "mobilisers"[All Fields] OR "mobilises"[All Fields] OR "mobilising"[All Fields] OR "mobilization"[All Fields] OR "mobilizations"[All Fields] OR "mobilize"[All Fields] OR "mobilized"[All Fields] OR "mobilizer"[All Fields] OR "mobilizers"[All Fields] OR "mobilizes"[All Fields] OR "mobilizing"[All Fields]) OR (("osteopathic physicians"[MeSH Terms] OR ("osteopathic"[All Fields] AND "physicians"[All Fields]) OR "osteopathic physicians"[All Fields] OR "osteopath"[All Fields] OR "osteopaths"[All Fields] OR "osteopathic"[All Fields]) AND ("musculoskeletal manipulations"[MeSH Terms] OR ("musculoskeletal"[All Fields] AND "manipulations"[All Fields]) OR "musculoskeletal manipulations"[All Fields] OR ("manual"[All Fields] AND "therapy"[All Fields]) OR "manual therapy"[All Fields])))

2-("manual therapy"[Title/Abstract] OR "osteopathic manipulation"[Title/Abstract] OR "mobilization"[Title/Abstract] OR "manipulation"[Title/Abstract] OR "mobilisation"[Title/Abstract] OR "osteopathic manual therapy"[Title/Abstract]) AND ("breathing exercises"[Title/Abstract] OR "inspiratory muscle training"[Title/Abstract] OR "respiratory muscle training"[Title/Abstract])

**EMBASE**

('manipulative medicine'/exp OR 'manipulation therapy' OR 'manipulation treatment' OR 'manipulative medicine' OR 'manipulative physical therapy' OR 'manipulative therapy' OR 'manipulative treatment' OR 'manual physical therapy' OR 'manual therapy' OR 'medicine, manipulative' OR 'therapy, manual') AND ('respiratory muscle training'/exp OR 'inspiratory muscle training'/exp OR 'breathing exercise'/exp OR 'breathing exercise' OR 'breathing exercises' OR 'breathing therapy' OR 'chest physical therapy' OR 'chest physiotherapy' OR 'exercise, breathing' OR 'exercise, respiratory' OR 'respiration exercise' OR 'respiration therapy' OR 'respiratory exercise' OR 'respiratory physiotherapy') AND ('maximal respiratory pressure'/exp OR 'maximal respiratory pressure' OR 'maximal respiratory pressures' OR 'forced expiratory volume'/exp OR 'fev' OR 'expiration index, forced' OR 'expiration volume, forced' OR 'fev 1' OR 'forced expiration index' OR 'forced expiration test' OR 'forced expiration volume' OR 'forced expiration, maximum' OR 'forced expiratory index' OR 'forced expiratory one second volume' OR 'forced expiratory volume' OR 'lung forced expiratory volume' OR 'lung maximal expiration volume' OR 'lung maximum expiratory volume' OR 'lung maximum expired volume' OR 'lung vital capacity' OR 'maximal expiration' OR 'maximal expiratory volume' OR 'maximal forced expiration' OR 'maximal inspiratory volume' OR 'maximal ventilation' OR 'maximum expiratory lung volume' OR 'maximum expiratory volume' OR 'maximum forced expiration' OR 'maximum lung capacity' OR 'one second forced expiratory volume' OR 'forced vital capacity'/exp OR 'fvc (forced vital capacity)' OR 'forced vital capacity')

**CINAHL**

( manual therapy or mobilization or manipulation or massage or osteopathy or osteopathic or chiropractic ) AND ( breathing exercises or breathing techniques or diaphragmatic breathing or deep breathing exercises or balloon blowing exercises ) AND ( spirometer or spirometry or incentive spirometer or incentive spirometry or lung function test )

( manual therapy or mobilization or manipulation or massage or osteopathy or osteopathic or chiropractic ) AND ( breathing exercises or breathing techniques or diaphragmatic breathing or deep breathing exercises or balloon blowing exercises )

**PEDro**

1. Abstract & Title: Manual Therapy AND Respiratory Rehabilitation. Method: clinical trial

2. Abstract & Title: Manual Therapy AND Inspiratory Muscle Training. Method: clinical trial

3. Abstract & Title: Manual Therapy AND Breathing Exercises. Method: clinical trial

4. Abstract & Title: Manual Therapy AND Respiratory Training. Method: clinical trial

**Annex 2.** Synthesis Funnel plot for forced expiratory volume during the first second parameter. Funnel plot aims to assess the existence of publication bias.

**Annex 3.** Meta-regression of intervention time of manual therapy and forced expiratory volume during the first second parameter (FEV_1_). The meta-regression approach uses regression analysis to determine the influence of selected variables (the independent variables) on the effect size (the dependent variable). The large bubbles, together with the line, indicate the relationship of our model, and the small bubbles indicate their position, the relationship in the map of the effect size on the intervention time, on the score in the variable of FEV_1_.

**Annex 4.** Synthesis Funnel plot for forced vital capacity parameter. Funnel plot aims to assess the existence of publication bias.

**Annex 5.** Meta-regression of intervention time of manual therapy and forced vital capacity (FVC) parameter. The meta-regression approach uses regression analysis to determine the influence of selected variables (the independent variables) on the effect size (the dependent variable). The large bubbles, together with the line, indicate the relationship of our model, and the small bubbles indicate their position, the relationship in the map of the effect size on the intervention time, on the score in the variable of FVC.

**Annex 6.** Synthesis Funnel plot for 6-minute walk test. Funnel plot aims to assess the existence of publication bias.

**Annex 7.** Meta-regression of intervention time of manual therapy and 6-minute walk test parameter (6-MWT). The meta-regression approach uses regression analysis to determine the influence of selected variables (the independent variables) on the effect size (the dependent variable). The large bubbles, together with the line, indicate the relationship of our model, and the small bubbles indicate their position, the relationship in the map of the effect size on the intervention time, on the score in the variable of 6-MWT.
